# Zebrafish orthologs of human muscular dystrophy genes

**DOI:** 10.1186/1471-2164-8-79

**Published:** 2007-03-20

**Authors:** Leta S Steffen, Jeffrey R Guyon, Emily D Vogel, Rosanna Beltre, Timothy J Pusack, Yi Zhou, Leonard I Zon, Louis M Kunkel

**Affiliations:** 1 Children's Hospital, Program in Genomics, Boston, MA, USA and Harvard Medical School, Department of Genetics, 300 Longwood Ave, Boston, MA 02115, USA; 2 Children's Hospital, Department of Hematology/Oncology, Boston, MA, USA and Harvard Medical School, Department of Genetics, 300 Longwood Ave, Boston, MA 02115, USA; 3 Howard Hughes Medical Institute, Children's Hospital, 300 Longwood Ave, Boston, MA 02115, USA

## Abstract

**Background:**

Human muscular dystrophies are a heterogeneous group of genetic disorders which cause decreased muscle strength and often result in premature death. There is no known cure for muscular dystrophy, nor have all causative genes been identified. Recent work in the small vertebrate zebrafish *Danio rerio *suggests that mutation or misregulation of zebrafish dystrophy orthologs can also cause muscular degeneration phenotypes in fish. To aid in the identification of new causative genes, this study identifies and maps zebrafish orthologs for all known human muscular dystrophy genes.

**Results:**

Zebrafish sequence databases were queried for transcripts orthologous to human dystrophy-causing genes, identifying transcripts for 28 out of 29 genes of interest. In addition, the genomic locations of all 29 genes have been found, allowing rapid candidate gene discovery during genetic mapping of zebrafish dystrophy mutants. 19 genes show conservation of syntenic relationships with humans and at least two genes appear to be duplicated in zebrafish. Significant sequence coverage on one or more BAC clone(s) was also identified for 24 of the genes to provide better local sequence information and easy updating of genomic locations as the zebrafish genome assembly continues to evolve.

**Conclusion:**

This resource supports zebrafish as a dystrophy model, suggesting maintenance of all known dystrophy-associated genes in the zebrafish genome. Coupled with the ability to conduct genetic screens and small molecule screens, zebrafish are thus an attractive model organism for isolating new dystrophy-causing genes/pathways and for use in high-throughput therapeutic discovery.

## Background

Muscular dystrophies are a heterogeneous group of genetic disorders characterized by loss of muscle strength and integrity. Common pathological hallmarks of the mammalian muscular dystrophies include the presence of necrotic muscle fibers, fiber size variation, centralized nuclei indicating fiber regeneration, inflammatory infiltrates, and replacement of muscle fibers by fat and connective tissue to varying degrees. However, muscular dystrophies differ in their age of onset, severity, the muscle groups affected, additional non-muscle phenotypes (such as reduced average IQ) and the genetic mode of inheritance (reviewed in [1]).

To date, 31 distinct muscular dystrophies have been described and 25 distinct genes have been causatively linked to these muscular dystophies [2]. The most common, Duchenne Muscular Dystrophy (DMD), accounts for the majority of dystrophy patients. DMD affects 1 in 3500 males and typically results in death by the third or fourth decade. The mutated gene in DMD, dystrophin, was identified in the 1980's [3] and its characterization has led to methods of genetic testing and a better understanding of dystrophic pathology. However, no cure has yet been identified. In addition, the causative genes remain unknown in several dystrophies and additional patients with unclassified dystrophy phenotypes. Finally, while several dystrophy-associated genes encode proteins that directly or indirectly interact, others, including the nuclear proteins (lamin A/C and emerin), and cytoplasmic proteins (TRIM32) have not yet been linked in a common pathway that would make apparent the cause of their dystrophic phenotype.

The small freshwater zebrafish, *Danio rerio*, has recently emerged as a promising model organism for the study of muscular dystrophies and other human diseases. Due to its small size, large numbers of offspring (50–350 per week), rapid development of the skeletal musculature, and transparency in embryonic/juvenile stages, zebrafish provide an excellent system for genetic screens to identify novel muscular dystrophy-causing genes and pathways. More recent experiments have also proven zebrafish a useful organism for drug screens using whole vertebrates, suggesting that identification of dystrophic zebrafish mutants may allow drug screens for muscular dystrophy therapeutics. (See [4-8] for zebrafish drug screens.)

The zebrafish *sapje *mutant was identified in 1996 with grossly normal muscle at 2 days post-fertilization (dpf) but decreased muscle organization and motility at 5 dpf [9]. The causative gene mutation was mapped to a nonsense mutation in dystrophin, suggesting conservation of this dystrophy-associated molecular pathway in fish [10]. Other studies have employed anti-sense morpholino or RNAi knockdowns to show similar dystrophy-like pathology and phenotypes when dystrophin (DMD/BMD), caveolin-3 (LGMD 1C), δ-sarcoglycan (LGMD 2F), or laminin α2 (MDC 1A) proteins are reduced, suggesting that this conservation may extend to other orthologs of human dystrophy-associated genes [10-16].

To aid in the further identification of zebrafish dystrophy mutants, we have interrogated current sequence databases to identify zebrafish orthologs of the known human dystrophy genes. Positioning these genes allows rapid candidate identification during genetic mapping of dystrophic zebrafish mutants and may allow prioritization of novel mutants – those with linkage to a genomic region containing no known dystrophy-associated ortholog. Due to the evolving nature of the Sanger Centre Zebrafish Genome assembly, we have also identified the BAC clone location of these genes. BAC sequences should allow more consistent local sequence information and easy updating when future genome alignments are released. This study has identified orthologous zebrafish transcripts for 24 out of 25 of the known human dystrophy-associated genes and 4 additional myopathy-related genes. Genomic positions have been identified for all 29 of these genes and BAC locations for 24. This genomic data suggests that at least two dystrophy genes are duplicated in the zebrafish genome. Localization of the closest mammalian gene neighbors also shows that syntenic relationships are conserved for 19 dystrophy- and myopathy-causing genes.

## Results and discussion

Mutations in 25 genes have been linked to 27 distinct forms of human muscular dystrophy (MD). In humans, these genes are distributed across 17 of the 23 chromosomes. Protein products of these genes position throughout the muscle fiber – from the extracellular matrix and sarcolemmal membrane to the sarcomere, the golgi, the cytoplasm, and the nucleus.

We surveyed the zebrafish GenBank database to identify putative orthologs of the 25 human muscular dystrophy-associated genes and four additional myopathy-associated genes. Results with a high degree of similarity and significant sequence coverage were confirmed by reciprocal blast into Genbank mammalian databases. This *in silico *approach identified orthologous transcripts for 23 out of 25 muscular dystrophy-associated genes and all 4 myopathy-associated genes within the Genbank database (Tables 1 and 2).

No orthologous transcript sequence was identified in the zebrafish Genbank database for the non-congenital MD gene, myotilin, or the congenital MD (CMD) gene, integrin alpha 7 (ITGA7). However, interrogation of Version 5 of the Sanger Centre Zebrafish Genome with human myotilin protein sequence identified a highly conserved ENSEMBL-predicted zebrafish myotilin transcript. Complete and contiguous sequence for this ENSEMBL-predicted myotilin was also found on a single BAC clone. However, the predicted transcript is no longer contiguous within Version 6 of the genome, suggesting that the current genomic alignment through this region may be incorrect. For integrin alpha 7 (ITGA7), a putative genomic and BAC location was identified by similarity to human ITGA7 over other integrins, though no transcript sequence has yet been identified. These data suggest that zebrafish have gene orthologs for all known human MD genes. In combination with mutant and morpholino data demonstrating zebrafish dystrophy phenotypes upon down-regulation of several MD gene orthologs, these data recommend the zebrafish as an excellent model organism for genetic screens to identify additional vertebrate MD-causing genes and pathogenic pathways.

### Genomic positions of zebrafish dystrophy orthologs

Genomic loci of zebrafish orthologs were identified in version 6 of the Sanger Centre Zebrafish Genome using the blastn algorithm with zebrafish RNA sequences. Locations were independently confirmed using the tblastn algorithm with human protein sequences. Human protein sequences were also used in case gene duplications were present but not reported in the EST database. Human sequences often returned several locations, sometimes correlating with related genes within a gene family. Additional loci were ruled out where possible by performing similar analyses with paralogs and/or by synteny with paralogs.

**Figure 1:**
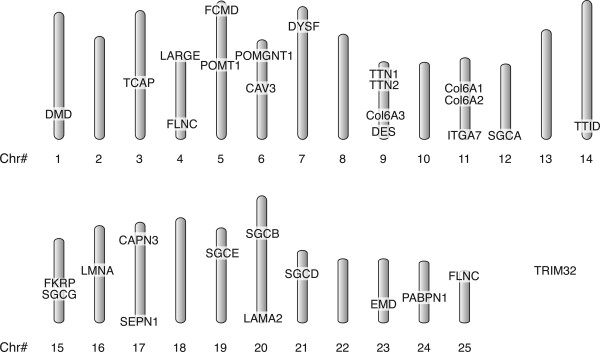
**Distribution of zebrafish muscular dystrophy orthologs**. Orthologs of the 25 muscular dystrophy-associated genes and 4 additional myopathy-associated genes were identified on 20 of the 25 zebrafish chromosomes by computer searches of the Sanger Centre Zebrafish Genome. Duplicate loci were found for FLNC and TTN orthologs. TRIM32 is located on an orphan scaffold that has not yet been integrated within the genome.

All 29 genes could be placed in whole or in part on Version 6 of the Sanger Centre Zebrafish Genome. TRIM32, responsible for Limb Girdle Muscular Dystrophy 2H (LGMD 2H), resides on an orphan scaffold that has not yet been integrated into the chromosomal organization of the genome. The remaining 28 genes are scattered across 18 chromosomes with the majority of chromosomes having only one dystrophy ortholog (Fig. 1). Only Chr 9 (Collagen 6A3, desmin, and duplicate titin genes) and Chr 11 (ITGA7 and two syntenic collagen genes) contain more than two dystrophy orthologs. It is interesting to note that there is currently no identified sex chromosome in zebrafish. Indeed, dystrophin and emerin, genes that reside on the human X chromosome, are found on different chromosomes in zebrafish, and characterization of the zebrafish dystrophin mutant, *sapje*, has demonstrated an autosomal recessive inheritance pattern.

Genomic loci identified in the Sanger Centre Database frequently showed non-contiguous organization of transcript sequences, suggesting that the genome is not yet correctly organized in these regions. Thus, BAC clone locations were identified within the Sanger Centre Zebrafish Clone Database to allow rapid updating of dystrophy ortholog positions as the genome assembly continues to evolve. Clone data was also used where possible to distinguish duplications due to genomic misalignments versus real duplications by determining if the associated clones overlapped. Using both zebrafish nucleotide and human protein sequences, at least partial BAC coverage was identified for 24 out of the 29 genes of interest.

### Genome loci verification

Version 6 of the Sanger Centre Zebrafish Genome contains better sequence coverage of the dystrophy-associated genes than the previous version, due in large part to use of physical data to integrate the clone sequence and whole shotgun method sequence (data not shown). Genomic positions could be found for all 29 genes, and 19 of these genes show conservation with humans of syntenic relationships with at least one neighboring gene, including TRIM32.

Though more complete than previous versions, Version 6 of the zebrafish genome is not yet entirely correct, since several transcripts appear split between distant genomic loci or have portions (usually corresponding to exons found singly on a BAC) multiply identified in close proximity on the genome. In particular, genes with repeat-rich or modular elements, like dystrophin and collagen 6A3, may be more difficult to align electronically, resulting in genomic sequences that do not agree with BAC sequence data. However, 24 of the transcripts were found with nearly complete coverage spanning one or more BAC clones which should provide better local sequence coverage until complete clone information has been incorporated into the genome assembly.

To test these data, we compared the *in silico *identification of genomic loci with those previously identified. To date, only four dystrophy-associated orthologs have been physically localized in the zebrafish genome. By radiation hybrid (RH) mapping using the T51 RH panel, researchers have mapped dystrophin to Chr 1 [13,17] and delta sarcoglycan to Chr 21 [12]. Similarly, caveolin 3 has been localized to Chr 6 [14]. Finally, a BAC walk between two genetic markers on Chr 9 identified and positioned titin in the interval [18]. All four positions agree with the data presented here from Version 6 of the Sanger Centre Zebrafish Genome. In addition, genetic mapping using polymorphic microsatellites within and flanking zebrafish titin has confirmed the duplication of zebrafish titin that was found *in silico *(data not shown).

To expand the set of genes for which we have physical position information, calpain-3 was located using radiation hybrid analysis with the T51 RH panel. *In silico *mapping places calpain-3 on Chr 17. However, RH mapping of calpain-3 places the orthologous zebrafish transcript on Chr 22 nearest to marker fa11a04.s1. To reconfirm computer-based findings, an independent analysis was performed and again returned Chr 17 as the most likely calpain-3 locus with synteny to neighboring genes. While BLAST analysis did identify other loci with some similarity to human calpains, none were located on Chr 22.

These data, the appearance of genomic duplications of genes in whole or in part, and the identification of non-contiguous transcripts in the genome suggest that the current Sanger Centre Zebrafish Genome still contains regions of misassembly, especially where continuity and singleness of transcripts is confirmed within the clone database. Nonetheless, locations for a greatly increased number of gene orthologs could be identified in Version 6 of the genome as compared with Version 5, suggesting improvement of the genome assembly over time (data not shown). In combination with the 80% success rate in the 5 genes with physical mapping data, this suggests a strong correlation between rough physical gene location and the current genome assembly.

### Gene duplications

Multiple distinct zebrafish transcripts were identified for each of four genes: Filamin C, emerin, dystrophin, and titin. For emerin, the two identified transcripts differ only by a single 7 basepair internal fragment, suggesting differential splicing, or mis-prediction of one of the transcripts. Both transcripts identified the same genomic and BAC locus, further suggesting a single gene locus. In the case of dystrophin, the multiple transcripts all appear to be partial sequences of the large dystrophin mRNA (> 14 kb in humans) [19], and position to Chr 1.

Two putative zebrafish FLNC transcripts were identified in Genbank that position to different genomic loci. Of these, only the FLNC predicted-transcript XM_693754 contains an exon highly orthologous to human exon 47, the conserved FLNC-unique region in mammals. A second FLNC transcript, XM_687344, is only a partial transcript and does not contain this exon. However, comparison of human FLNC exon 47 with the zebrafish genome identified a second locus for this exon immediately following the genomic locus of XM_687344, suggesting that a full transcript sequence would identify this gene as FLNC. Filamin C (FLNC) appears to be a true duplication, with transcripts divergent at the nucleotide and protein levels.

Titin, which codes for an enormous mRNA in humans (> 82 kb) [20], shows multiple transcripts due to its length, as well as an apparent gene duplication event. Duplicated loci were found in head-to-tail juxtaposition with genes divergent at both the nucleotide and protein levels. Due to the large number of titin transcripts, only two transcripts from each gene locus have been listed. Two additional genes, dysferlin and desmin, may also have genomic duplications, identified by multiple zebrafish genomic loci orthologous to the human protein sequences. While many of these loci were ruled out due to closer homology with other human proteins within the gene families, not all additional loci could be eliminated.

Studies of the Hox gene clusters in fish suggest a full genome duplication event in ancestral teleost lineages after the divergence of ray-finned fish (from which zebrafish derive) and lobe-finned fish (from which mammals derive) [21]. Further comparative genomics studies report that at least 20% of gene duplicates have been maintained in zebrafish, often by divergence of regulation between the duplicate loci that imposes an evolutionary constraint on both genes [22]. Of the zebrafish gene orthologs in this study, however, we find that only two genes show strong evidence of duplicate gene maintenance – titin and FLNC – with at most four gene duplications suggested by the genomic sequence (including dysferlin and desmin). Further, the juxtaposition of duplicate titin loci strongly suggests a tandem gene duplication event *after *the teleost ancestral genome duplication.

Thus, at least one and at most 3 of the 29 genes studied (3–10%) show evolutionary maintenance of duplicate gene sequences from the whole genome duplication event, below the 20% previously reported [22]. Given the widespread distribution of these genes (Fig. 1), it is unlikely that the absence of dystrophy gene duplications is due to lack of duplication of a specific chromosomal region, or to secondary loss of a specific chromosomal region after polyploidization. It is also unlikely that the low number of dystrophy gene duplications in zebrafish is the result of an overall detrimental affect of duplicate copies of these genes since paralogs of many dystrophy genes are found in both mammals and fish. While it is quite possible that all existing duplicates were not identified in this study, it is also possible that these genes may evolve more slowly, preventing divergence of duplicate loci that would subject both to evolutionary constraint.

## Conclusion

To aid in the development of zebrafish as a suitable candidate for genetic screens for dystrophy-causing mutations and to create a genomic map of dystrophy-associated zebrafish genes, we searched existing zebrafish sequence databases to identify zebrafish orthologs of dystrophy-causing genes. Using Genbank and Sanger Centre databases, 28 out of 29 genes studied showed identifiable ortholog transcripts. These data suggest that zebrafish may express muscle genes orthologous to those previously shown in mammals to be required for normal muscle maintenance and/or regeneration. Genomic loci were also identified for all 29 genes (though one, TRIM32, is currently located on an orphan scaffold). Comparison of *in silico *ortholog mapping with published physical mapping confirms that the current genome and *in silico *techniques were able to identify correct chromosomal locations for at least 4 genes out of 5 genes with available positional information. Only 3–10% of dystrophy gene duplicates appear to have been maintained since the teleost genome duplication, fewer than other gene groups studied in fish, indicating that the dystrophy-related genes may be slow to evolve independent functions or regulation. These data should aid in the genetic mapping of zebrafish dystrophy mutants, creation of mutant lines for high-throughput testing of dystrophy therapies, and identification of novel dystrophy-causing genes.

## Methods

### Computer identification of orthologous zebrafish ESTs

For each gene (Table 1 and Table 2), human transcript sequences (starting with NM) and human protein sequences (starting with NP) were identified in NCBI databases [23]. Zebrafish ESTs orthologous to the human protein sequences were identified by BLAST into the NCBI zebrafish nr database using the tblastn algorithm. Responses were prioritized by percentage similarity and amount of coverage. Where more than one reasonable candidate EST was returned, all such ESTs were reciprocally compared with mammalian sequences in NCBI (nr database) using the tblastx algorithm to determine which one was most similar to the mammalian gene being studied. Sequences starting with NM representing known EST sequences were preferred. Predicted sequences (starting with XM) were used only when they showed high percentage similarity to mammalian sequences and when no other highly correlated zebrafish ESTs were returned. In some cases, zebrafish ortholog candidates could still not be distinguished and all such candidates were noted and pursued for the following identification steps. Where more than one human isoform is known for a given gene, all isoforms were independently queried against zebrafish databases as above. In no case, however, did different isoforms of a single human gene identify disparate zebrafish genes.

### Computer identification of genomic location

Zebrafish ESTs were then compared with the current zebrafish genome assembly, Zv6, in the Wellcome Trust Sanger Institute databases as of April 2006. To identify genomic locations of zebrafish ortholog ESTs, the Ensembl blast program [24] was used with the blastn algorithm and "Near exact match" parameters. Returned hits were ranked by e value and assessed for transcript coverage. Note that percentage of sequence identity was typically > 95% over short stretches (likely corresponding to exons). Where more than one location had similar levels of coverage and sequence identity, all such locations are noted. To confirm genomic loci (or if no zebrafish EST was identified), human protein sequences were compared with the genome using a tblastx algorithm and the parameter "Allow some local mismatch". Multiple loci were frequently identified with the human protein, but could often be ruled out based on a closer orthology to other genes within a gene group (using the analyses methods herein).

### Computer identification of position on sequenced clones

Because the genome assembly is still not complete and certain regions may be misaligned, we also identified the clone locations of zebrafish genes where possible. Zebrafish ESTs were compared with finished and unfinished clone sequences using the Sanger D. rerio Blast Server [25]. The blastn algorithm was used with a filter for low complexity regions and Repeatmasker to mask short repeat sequences. Returned sequences were ordered by e value and analyzed for coverage and exon breaks corresponding to those seen in genomic locations. All finished clone sequences with complete coverage are listed. Unfinished (incompletely sequenced) clones are noted only where there was no reasonable alignment with a finished clone. In the case where a gene spans more than one clone, clones are noted with plusses between them. Again, loci were confirmed by homology to human protein sequences using the tblastn algorithm (without Repeatmasker).

### Determination of synteny

Neighboring genes and their orientations with respect to the human gene of interest were determined using NCBI Entrez GeneView. In many cases, closest neighbors were predicted or non-coding RNAs. Non-coding RNAs were not used. Some predicted genes did retain syntenic relationships and are listed. Where neighboring predicted genes were not found in the zebrafish genome, the closest known coding gene was used instead. Genomic loci for neighboring genes were determined as above, using the human protein sequence and tblastn algorithm in either the Zebrafish Genome or in the Clone Database.

### Radiation hybrid mapping

Primers were designed to calpain-3 sequence NM_001004571 and used in PCR reactions with the zebrafish T51 radiation hybrid panel as previously described [26,27]. SAMapper was used to obtain LOD scores and map distances to known zebrafish markers [28]. Primers used were:

CAPN3 (Forward): 5'- CACTAGTGTCACAGGCAGCGTTTC-3'

CAPN3 (Reverse): 5'- GTTGCCGTCCATCATGAGCTTTGAG-3'

## Authors' contributions

LSS identified ortholog sequences and genomic map locations and drafted the manuscript. JRG and LMK conceived of the project and assisted in drafting the manuscript. JRG also performed initial sequence searches. EDV and TJP participated in initial sequence searches. RB and YZ performed the radiation hybrid mapping of calpain-3 and assisted in project design. All authors read and approved the final manuscript.

## Figures and Tables

**Table 1:** Zebrafish orthologs of human muscular dystrophy genes and their genomic locations.

					**Fish Genomic Location**				**Fish BAC Loc**			
							
**Gene**	**Symbol**	**Associated Disease**	**Fish EST**	**Notes**	**Scaffold location**	**Clone Location**	**Gene location**	**Notes**	**BAC Name**	**Notes**	**Synteny**	**Notes**
Calpain-3	**CAPN3**	LGMD2A	NM_001004571		2601	NA-BX322589	Chr17 62.6 Mb and 63.1 Mb	By blasts and synteny, appears split,. The first ~ 1550 nt has no corresponding BAC.	N/A – zC283F6	Human CAPN3 matches well with zK12H9	Yes	Syntenic with GANC on genome and ZFP106 on genome and BAC (zC283F6)

Caveolin 3	**CAV3**	LGMD1C, hyperCKemia, Rippling muscle disease	NM_205738		879	BX664752	Chr6 33.2 Mb	Organized in 2 exons, similar to human CAV3	zKp111E5		On one side	Syntenic with OXTR on BAC and genome.

Dystrophin	**DMD**	Duchenne MD, Becker MD	XM_678461, XM_678362, XM_678552, partial	All three are partial transcripts, but in order, cover most of the DMD coding region	42	BX004756, CT033808	Chr1 9.6 Mb-9.4 Mb	Duplications within gene likely incorrect. Additional partial sequence located on Chr1 scaffold 49 at 10.5 Mb	zC59A4, zC274B7	Transcripts span these two overlapping BACs	No	

Dysferlin	**DYSF**	LGMD2B, Miyoshi Myopathy, Distal myopathy with anterior tibial onset	XM_684324	Many transcripts are similar to dysferlin, but this is the only one that aligns closer to dysferlin (rather than myoferlin or otoferlin) on reciprocal blast	1155	CR847843	Chr7 83.3 Mb-83.5 Mb	Human dysferlin also identifies Chr12 (BC063743, likely fish myoferlin) and Chr13 (XM_682373, similar to myoferlin, dysferlin, and otoferlin)	zKp78E10 – bZ50C18	The first BAC contains the 5' ~ 1/7th while the second BAC overlaps and gives coverage to the transcript end	No	Flanking genes are on Chr7 but not in the same region or on the same scaffold as dysferlin

Emerin	**EMD**	Emery-Dreifuss MD	XM_685843, XM_549369	Identical except for a single 7nt internal fragment. Likely alternatively spliced variants. Poor homology to mammalian emerin.	3352	No data	Chr23 18.96 MB	Duplication of last 4/5 of transcript on Chr7 39.54 Mb, scaffold 1085. No synteny with Chr7.	zC133L21	Identical matches on unfinished BACs zK233H12, zK181F15, and zK93L1.	Yes	RPL10 and FLNA are at 19.1 Mb and 19.0 Mb, on Chr23 Both are syntenic on BACs.

Fukutin related protein	**FKRP**	MDC1C, LGMD2I	XM_695011, partial		2206	No data	Chr15 26.6 Mb		zK31C13		Yes	STRN4 and SLC1A5 are syntenic on the genome and the BAC.

Lamin A/C	**LMNA**	Emery-Dreifuss MD, LGMD1B, CMD1A, etc.	NM_152971		2371	CR848742	Chr16 37.8 Mb		zK181C1		No	Flanking genes are not syntenic with each other, either

Myotilin	**TTID**	LGMD1A, myofibrillar myopathy	None found	Closest match is Zv5 predicted transcript ENSDARG015348	1999	CT573287	Chr14 12.2 Mb & 10.8 Mb	ENSDARG015348 split between two loci in Zv6. Genome incorrect.	zK101K8	Complete and contiguous BAC coverage	No	Flanking genes are not syntenic with each other, either

Sarcoglycan alpha	**SGCA**	LGMD2D	XM_680178	Close homology with SCGE	1664	BX548040	Chr12 870 Kb		zC190L11		On one side	Syntenic with Col1A1 on both genome and BAC

Sarcoglycan beta	**SGCB**	LGMD2E	NM_001034973	First half of transcript aligns with full length of human SGCB	2974	CT583700	Chr20 59.8 Mb	Second half of EST is located on Chr25 1.3 Mb (Scaffold 3566)	zC253J24		Yes	Both genes syntenic on genome and BAC

Sarcoglycan delta	**SGCD**	LGMD2F, CMD1L	NM_001001816		3106	BX294656	Chr21 39.0–38.7 Mb		zC238M13	First 300nt located on zK189O20	On one side	Syntenic with MRPL22

Sarcoglycan gamma	**SGCG**	LGMD2C	NM_001003748		2184	BX927291	Chr15 20.2 Mb	Incomplete coverage. Duplicate exon also on scaffold 2184 but not on BAC.	zC261A10	Complete coverage	On one side	Syntenic with SACS on both genome and BAC

Telethonin	**TCAP**	LGMD2G, Dilated cardio-myopathy (CMD1N)	XM_679011		371	CR387996	Chr3 19.9 Mb	Human TCAP also identifies a locus on Chr16 scaffold 2377 but coverage is less complete and exons are not contiguous	zK183N6		No	

Tripartite Motif-containing protein 32	**TRIM32**	LGMD2H	XM_686142	Human TRIM32 only has one coding exon.	NA688	No data	No data	Coding sequence on scaffold NA688. Putative 5' UTR exons are located in duplicate on Chr8, scaffold 1244	None	Putative 5' UTR exons are located on zK72L14 & zK65O14	Yes	ASTN2 spans the both the human and the zebrafish TRIM32 loci on Scaffold NA688

Titin	**TTN**	LGMD2J, Tibial MD, Hereditary Myopathy with early respiratory failure	XM_679005 (TTN2, partial), XM_678144 (TTN1, partial)	Locus is duplicated. Only partial transcripts available	3186	BX640499, BX571737, BX640465	Chr9 41.8 Mb-42.2 Mb	Locus duplications are in tandem. Duplicate genes are divergent in sequence and likely to be true duplicates.	zKp67D2, dZ258D18, zK190I10, dZ249N21, zC198B21	BACs overlap to cover the entire titin locus.	Yes	Syntenic with FLJ39502 and FKBP7 on genome and BAC.

MD-Muscular Dystrophy, LGMD-Limb Girdle Muscular Dystrophy, CMD-Congenital Muscular Dystrophy, nt-nucleotides.

**Table 2:** Zebrafish orthologs of human congenital muscular dystrophy and selected myopathy genes and their genomic locations. Genes associated with both non-congenital and congenital muscular dystrophies are in Table 2-1.

					**Fish Genomic Location**				**Fish BAC Loc**			
							
**Gene**	**Symbol**	**Associated Disease**	**Fish EST**	**Notes**	**Scaffold location**	**Clone Location**	**Gene location**	**Notes**	**BAC Name**	**Notes**	**Synteny**	**Notes**
Collagen 6A1	**Col6A1**	Bethlem myopathy, Ullrich CMD	XM_693161, partial		1607	CR925698	Chr11 35.3 Mb		zK287I12		On one side	Syntenic with Col6A2 on genome and BAC.

Collagen 6A2	**Col6A2**	Bethlem myopathy, Ullrich CMD	XM_691072	The first 320 bases are likely not part of this transcript.	1607	CR925698-BX323597	Chr11 35.2 Mb	The first 320 bases are located in multiple places on other chromosomes	zK287I12 – zC227N13	The first 320 bases are located on zC184B9.	On one side	Syntenic with Col6A1 on genome and BAC.

Collagen 6A3	**Col6A3**	Bethlem myopathy, Ullrich CMD	XM_679796	XM_687365 is also orthologous to mammalian Col6A3, but is more similar to a second predicted Col6A3 mammalian locus.	1361-1360	No data	Chr9 19.0 Mb and 15.0 Mb	The beginning is located on scaffold 1361, the repeating middle elements are on both scaffolds, and the end is on 1360. Note that the genomic locus may be misorganized.	zC5M6	Unfinished BAC covers entire transcript on various fragments	Yes	Syntenic with MLPH on Chr9 and with COPS8 on Chr9 and clone zC5M6.

Desmin	**DES**	DCM1, CMD1I, several skeletal and/or cardio-myopathies	NM_130963		1342	No data	Chr9 7.3 Mb	Several loci are orthologous to human desmin. Most ruled out due to closer homology with other proteins. Additional loci on Chr20 (scaffold 2945), and Chr13 (scaffold 1885) could not be ruled out and may be duplications.	None	Homologous sequences were found, but none were near-exact matches to the zebrafish transcript sequence.	No	Chr9 locus is not syntenic with the other desmin-like genes, either.

Fukutin	**FCMD**	Fukuyama CMD	XM_686729, partial		792, 793	CR753888, CT027618, BX072578	Chr5 78.4–79.0 Mb	Full match on the first two clones, partial match on the third. Likely a genomic misalignment.	zC286A10, zC154E10	Full coverage of the partial transcript on both	On one side	FSD1CL is syntenic on both genome and BAC.

Filamin C	**FLNC**	Myofibrillar myopathy	XM_693754, XM_687344, partial	Duplicated. Divergent nucleotide sequences. First contains the Human FLNC unique region. Second transcript is only partial.	505, 3643	AL954190, No data	Chr4 7.5 Mb, Chr25 32.9 Mb	Human FLNC unique region is not part of XM_687344, but is located immediately after it on Chr25.	zC284B12, zK3006	Both BACs match XM_693754. No BACs for XM_687344	On one side	Chr4 locus not syntenic, though flanking genes are elsewhere on Chr4. Partial NAG6 matches on Chr25.

Integrin Alpha 7	**ITGA7**	CMD with integrin deficiency	None found	Closest EST is a closer match to mammalian ITGA6	1560	No data	Chr11 2.5 Mb	Location identified using human ITGA7 only	zC245G15	Used human ITGA7	No	Flanking genes are not syntenic with each other, either

Acetyl-glucosaminyl-transferase-like protein	**LARGE**	MDC1D	NM_001004537	LARGE1B (NM_001004538) is highly orthologous.	570	No data	Chr4 39.4 Mb	LARGE1B located on Chr18, scaffold 2725, clone BX908385.	None	LARGE1B located on both zC282N12 & zC206G24	No	The closest flanking genes are predictions

Laminin alpha 2	**LAMA2**	Merosin-deficient CMD	XM_694983	Partial, predicted	2875	No data	Chr20 3.8 Mb	Aligns with LAMA2 predicted transcripts GenScan01065 and FGENESH78171	None		On one side	Syntenic with ARHGAP18, but NOT the highly similar LAMA1 locus (on Chr24)

Polyadenylate-binding protein, nuclear 1	**PABPN1**	Oculo-pharyngeal MD	BC079522	NM_213259 also matches but diverges over the 3' non-coding end. NM_213259 3' end is discontinuous with its 5' end on the genome and BACs and may not represent a real transcript.	3471	BX294113 and CT583644	Chr24 21.4 Mb and 21.6 Mb	Duplication on Chr24 clones is likely due to genomic misalignment since clones overlap in the Sanger fingerprinted contigs.	zKp73G8		No	SLC22A17 is located on Chr24, but not in the same region.

Protein O-Mannose Beta-1,2-N-Acetyl-glucosaminyl-transferase	**POMGNT1**	Muscle-eye-brain (MEB)	BC097123		985	No data	Chr6 69.0 Mb		zK170G13, zC156B18	Sequencing of first BAC is unfinished	On one side	Syntenic with TSPAN1 on both genome and BAC

Protein-O-mannosyl-transferase 1	**POMT1**	LGMD2K, Walker Warburg syndrome	XM_693177		723	BX511209 and No data	Chr5 56.2 Mb & 56.3 Mb	Split between 3 loci. Exons 1–3 at first location, exons 3–17 at second location. Exons 17–22 potentially on Chr17 at 37.47 Mb.	zC129A6	Covers only first 3 exons. No matches for other exons.	No	

Sarcoglycan epsilon	**SGCE**	Myoclonic dystonia	NM_001002594	Close homology with SCGA	2827	BX640469	Chr19 41.07 Mb		zK104M9		On one side	Syntenic with CASD1 on both genome and BAC

Selenoprotein N, 1	**SEPN1**	Rigid spine MD1 (RSMD1), Multiminicore disease	NM_001004294		2451	BX323794 & R626962	Chr17 1.8 Mb & 2.3 Mb	Duplication likely due to genome misalignment since the BACs overlap. Both clones have full transcript coverage.	zC247C16, zC15D5	BACs overlap, suggesting that the genomic duplication is a misalignment.	On one side	Syntenic with FAM54B on genome and BAC

CMD/DCM-Congenital Muscular Dystrophy, MD-Muscular Dystrophy, nt-nucleotides.
